# Relationship between self-reported pain, pain threshold, pain catastrophization and quality of life in patients with TMD

**DOI:** 10.4317/jced.59480

**Published:** 2023-01-01

**Authors:** Marcella-Santos Januzzi, Clóvis-Lamartine-de Moraes-Melo Neto, Amália Moreno, Emerson-Gomes dos Santos, Fernanda-Pereira de Caxias, Emily-Vivianne-Freitas da Silva, Flávia-Florêncio de Athayde, Augusto-Henrique-de Souza Volce, Alana-Semenzin Rodrigues, Juliana Dela Líbera, Karina-Helga-Leal Turcio

**Affiliations:** 1 Departamento de Materiais Odontológicos e Prótese, Universidade Estadual Paulista “Júlio de Mesquita Filho” (UNESP), Faculdade de Odontologia, Araçatuba, Brasil; 2 Departamento de Cirurgia Oral, Patologia e Clínica Dental, Universidade Federal de Minas Gerais (UFMG), Faculdade de Odontologia, Belo Horizonte, Brasil; 3 Departamento de Administração, Universidade Federal de São Paulo (UNIFESP), Osasco, Brasil; 4 Departamento de Produção e Saúde Animal, Universidade Estadual Paulista “Júlio de Mesquita Filho” (UNESP), Faculdade de Medicina Veterinária, Araçatuba, Brasil

## Abstract

**Background:**

The aim of this study was to verify if there is a relationship between self-reported pain, PPT (pressure pain threshold) of the masseter, temporal and sternocleidomastoid muscles, pain catastrophizing and quality of life in patients with TMD (temporomandibular disorder) of muscular origin.

**Material and Methods:**

Ninety-seven patients with muscular TMD (TMD group) and 97 asymptomatic (control group) were included in the study. The evaluation methods used were: 1) Self-reported pain was assessed using the Visual Analogue Scale (VAS) for questions 7, 8 and 9 of the RDC/TMD Axis I questionnaire; 2) The PPT assessment was performed using a digital algometer on the masseter, temporal, and sternocleidomastoid muscles (both sides); 3) Pain catastrophizing was assessed using the PCS (Pain Catastrophizing Scale); and 4) Oral health-related quality of life was assessed using the OHIP-14 (Oral Healthy Impact Profile-14). Data were submitted to Spearman correlation and logistic regression (*p*<0.05).

**Results:**

There were significant positive correlations between self-reported pain (VAS-Q7, VAS-Q8 and VAS-Q9), pain catastrophizing (PCS-Helplessness, PCS-Magnification, PCS-Rumination and PCS-Total) and quality of life (OHIP-14) (*p*<0.05). There was a significant negative correlation of self-reported pain (VAS-Q8) with PPT of the temporal (left) and sternocleidomastoid (both sides) (*p*<0.05). The rumination and magnification domains increased the chance of high self-reported pain in all situations (VAS-Q7, VAS-Q8 and VAS-Q9) (*p*<0.05). The helplessness domain only increased the chance of high self-reported pain for VAS-Q8 (*p*<0.05). The presence of TMD of muscular origin, high self-reported pain (VAS-Q7) or pain catastrophizing increased the chance of a low quality of life in relation to the control group (*p*<0.05). In addition, the reduction in sternocleidomastoid PPT increased the chance of poor quality of life (*p*<0.05).

** Key words:**Myofascial pain syndromes, pain catastrophizing, myalgia, quality of life, surveys and questionnaires, temporomandibular joint disorders.

## Introduction

The most common causes of chronic pain in the orofacial area are temporomandibular disorders (TMDs) (1). The TMDs are defined as a set of signals and symptoms that affect the temporomandibular articulations, masticatory muscles (e.g., masseter and temporal), or both (2,3). For many years, occlusal factors were attributed as the main etiological factors of TMDs, and they are still the subject of scientific debates (4,5). Currently, psychological factors (e.g., depression, anxiety, and psychological stress) and the presence of parafunctions are included in the list of etiological factors for the development, continuity and intensification of this pathology (6).

Pain in the jaw muscles is the most common type of pain in patients with TMD (3). This pain is usually chronic and includes the features of pain at rest and exacerbated pain during jaw functions such as biting, chewing, and yawning (3). For this type of TMD (muscular origin), the painful area is often tender to palpation, indicating a reduced pain threshold (7). Algometry is a reliable method used to quantify the degree of soft tissue sensitivity (7,8). This method measures the pressure applied to the muscle with a small rubber tip (8). The force that causes pain during pressure is called the pressure pain threshold (PPT) (8).

Pain catastrophizing is a cognitive factor represented by an exaggeration of the perceived threat of pain sensation (9). It is described in terms of a multidimensional construct including rumination (not being able to direct

attention away from pain), magnification (worry or exaggeration of the seriousness of something”), and helplessness (“feeling nothing can be done to reduce the pain) (1,10). Pain catastrophizing clearly plays a role in the suffering of patients with orofacial pain, causing these patients to use health services more frequently (11). In addition, pain catastrophizing is related to fear of pain due to movement (kinesiophobia), affecting the ability to perform tasks such as eating, chewing, and communicating (10).

People with TMD often experience tension or pain in the neck (12). The sternocleidomastoid is a neck muscle that is considered one of the controllers of the patient’s head position, for example, during mastication (12,13). Thus, in studies that assess pain in the masticatory muscles due to TMD, it is also important to assess the neck muscles.

The aim of this study was to verify if there is a relationship between self-reported pain, PPT of the masseter, temporal and sternocleidomastoid muscles, pain catastrophizing and quality of life in patients with TMD of muscular origin.

## Material and Methods

-Groups

This study was approved by the Ethics Committee of the Araçatuba Dental School (São Paulo State University [UNESP-FOA] - Certificate of Presentation of Ethical Appreciation: 69013417.5.0000.5420).

TMD group - the recruitment of individuals for this group was carried out at the Center for TMD Diagnosis and Treatment of UNESP-FOA. Control group - the recruitment of individuals for this group was carried out in the dental care clinics of UNESP-FOA. Control group was defined by the pairing technique according to relevant variables (sex and age) obtained through the “propensity score”. The recruitment period for both groups was from 2016 to 2019.

-Inclusion criteria

[1] 18 years or older.

[2] To be able to understand questions.

[3] To be fully or partially toothed (maximum of 2 missing dental crowns per arch).

[4] For the TMD group, individuals whose main complaint was pain due to TMD in the temporal and masseter muscles associated with pain in the sternocleidomastoid muscle for at least 3 months (2,14).

[5] For the control group - asymptomatic individuals.

-Exclusion criteria

[1] Serious diseases (e.g., trigeminal neuralgia, tumors, neurological diseases, degenerative diseases, psychiatric problems, narcolepsy and neuropathic pain).

[2] Complete denture and removable partial denture wearers.

[3] Person diagnosed with dental malocclusion; overjet and overbite greater than 6 mm; and cross bite.

[4] Use of medications that can interfere with muscle activity and pain, such as anxiolytics, antidepressants, and opioids.

[5] Frequent consumption of alcohol.

[6] Use of illicit drugs.

[7] Previous history of temporomandibular joint surgery.

[8] Pregnancy.

[9] Presence of primary headaches.

[10] When the main complaint was pain due to TMD of joint origin.

[11] Those who did not want to participate in this research.

-Assessment of oral health-related quality of life, pain catastrophizing and self-reported pain

Oral health-related quality of life was assessed using the Oral Healthy Impact Profile-14 (OHIP-14) questionnaire (3,15).

Pain catastrophizing was assessed using the Portuguese version of the Pain Catastrophizing Scale (PCS) (16). The PCS is a self-administered questionnaire that consists of 13 items to assess catastrophizers (16). It is divided into three domains: “helplessness”, “magnification”, and “rumination” (16).

Self-reported pain was assessed using the Visual Analogue Scale (VAS) for questions 7, 8 and 9 of the RDC/TMD Axis I questionnaire***, replacing the period of “6 months” with “3 months” (2).

***RDC/TMD Axis I questionnaire.

Question 7 (VAS-Q7) - How would you rate your facial pain on a 0 to 10 scale at the present time, that is right now, where 0 is “no pain” and 10 is “pain as bad as could be”?

Question 8 (VAS-Q8) - In the past 3 months, how intense was your worst pain rated on a 0 to 10 scale where 0 is “no pain” and 10 is “pain as bad as could be”?

Question 9 (VAS-Q9) - In the past 3 months, on the average, how intense was your pain rated on a 0 to 10 scale where 0 is “no pain” and 10 is “pain as bad as could be”? [That is, your usual pain at times you were experiencing pain]).

-Pressure pain threshold assessment using algometry

PPT assessment was performed bilaterally using a digital algometer (Wagner Instruments, Model FDI, USA) on the masseter, temporal (anterior part) and sternocleidomastoid muscles. In addition, this test was also performed on the flexor pollicis brevis muscle (right side) (control site).

For the temporal muscle (anterior part), algometry was performed on its center (7).

For the masseter muscle, pressure was applied to its center at a location that was ~50% of the distance from the zygomatic arch and the angle of the mandible (17).

For the sternocleidomastoid muscle, the operator held this muscle to perform algometry on its middle third (8).

Patients were instructed to raise their hand as soon as the sensation of pressure became painful (7). Three measurements were performed for each muscle with a 3-minute interval between measurements. Subsequently, a mean of the 3 measurements was obtained. The unit of measurement for the PPT test was Kgf/cm2.

-Statistical Analysis

Descriptive statistical analysis was performed with demographic data and variables of interest for this study using IBM SPSS Statistics (Version 22.0, Statistical Package for Social Science, USA).

The Wilcoxon test and the Mann-Whitney U test were used to evaluate the results of the PPT test.

The Spearman correlation test verified the correlations between the variables of this study.

Logistic regression was performed using R software (version 3.5.3, R Foundation for Statistical Computing, Austria). The models were the absence or presence of PCS domains in pain assessments. In addition, for quality of life, the models were the absence or presence of high self-reported pain, high PPT and PCS-TOTAL in quality of life assessments. All models estimated odds ratios with upper and lower confidence limits (95% confidence interval).

The *p* values less than 0.05 were considered statistically significant.

## Results

Two hundred and eighteen individuals were selected for this study (TMD group = 113 and control group = 105). After applying the inclusion and exclusion criteria, 97 patients remained in TMD group and 97 volunteers remained in the control group. The reasons for excluding 24 participants were as follows: For the DTM group, 16 people were excluded (1- psychiatric problem; 2- complete denture wearers, 8- use of benzodiazepines; 1- pregnancy; and 4- use of antidepressants); and for the control group, 8 people were excluded (3- complete denture wearers, 3- use of benzodiazepines; and 2- use of antidepressants).

Table 1 shows the mean results for age and sex in each group, and the medians of the results of the questionnaires used. TMD group showed a predominance of women. The TMD group showed higher medians for PCS (PCS-Helplessness, PCS-Magnification, PCS-Rumination and PCS-Total) and OHIP-14 than the control group (Table 1).

**Table 1:**
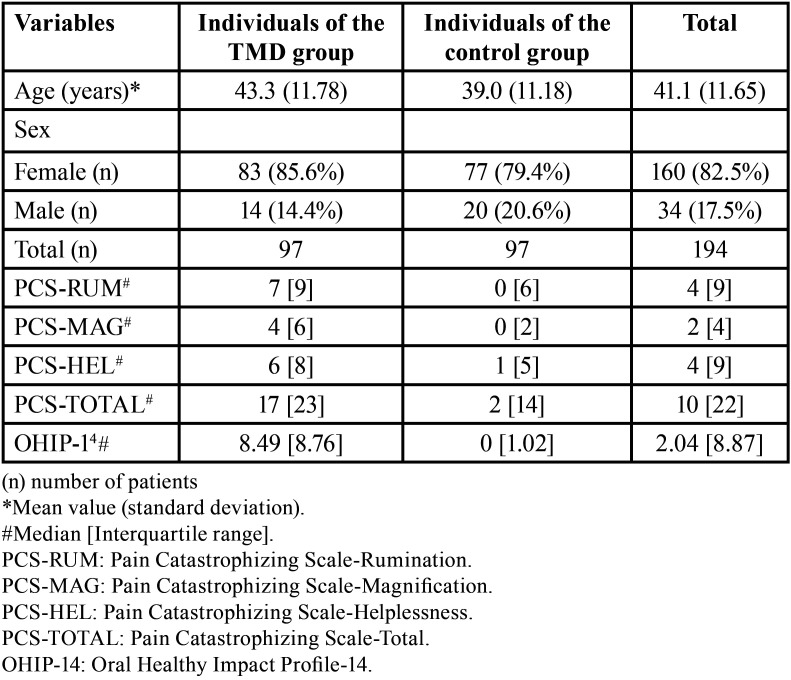
Descriptive data of study participants.

**Figure 1:**
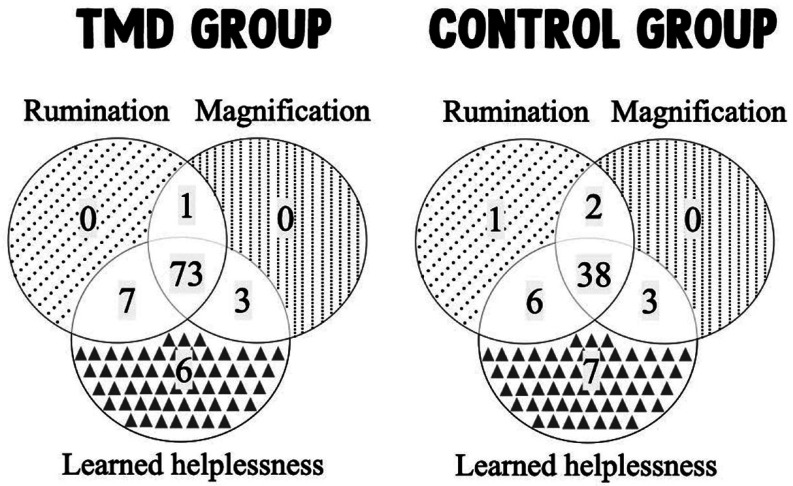
Venn diagrams showing the number of patients with the Pain Catastrophizing Scale domains.

For the PCS domains, 73 patients in TMD group presented all domains, while 11 patients presented 2 domains, and 6 patients presented 1 domain (Fig. 1- TMD group diagram). For the PCS domains, the control group had 38 patients with all domains, while 11 patients had 2 domains and 8 patients had 1 domain (Fig. 1 – Control group diagram).

**Figure 2:**
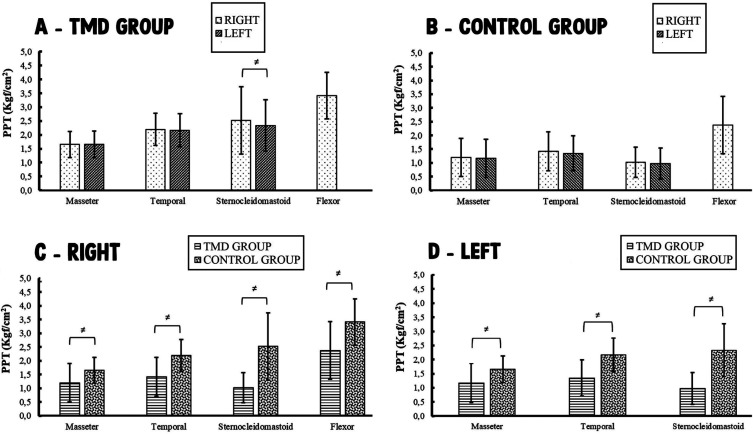
Pressure pain threshold test results (Kgf/cm2). ≠ represents a statistically significant difference (*p*<0.05); Wilcoxon test / Mann-Whitney U test. Flexor - Flexor pollicis brevis muscle. A - TMD group: Comparison of the right side with the left side in individuals with TMD of muscular origin. B - Control group: Comparison of the right side with the left side in asymptomatic individuals. C - Comparison of the right side of TMD group with the right side of control group. D - Comparison of the left side of TMD group with the left side of control group.

For the PPT test, when comparing the right side with the left side of each muscle studied in each group, there was a significant difference between sides only for the sternocleidomastoid muscle in TMD group (*p*=0.039 [2.52 Kgf/cm2 right; 2.34 Kgf/cm2 left]) (Fig. 2 A,B). When comparing TMD group with control group, based on the same muscle and side, it is possible to verify that individuals with TMD had significantly lower PPT values than individuals without TMD for all muscles (both sides) (*p*<0.05) (Fig. 2 C,D). The extra-trigeminal area evaluated showed significantly lower PPT value in TMD group compared with control group (*p*<0.05) (Fig. 2C).

The Spearman test results are shown in Table 2 for TMD group. There were significant positive correlations between self-reported pain (VAS-Q7, VAS-Q8 and VAS-Q9), pain catastrophizing (PCS-Helplessness, PCS-Magnification, PCS-Rumination and PCS-Total) and quality of life (OHIP-14) (*p*<0.05). In addition, there was a significant negative correlation of self-reported pain (VAS-Q8) with PPT of the temporal (left) and sternocleidomastoid (both sides) (*p*<0.05) (Table 2).

**Table 2:**
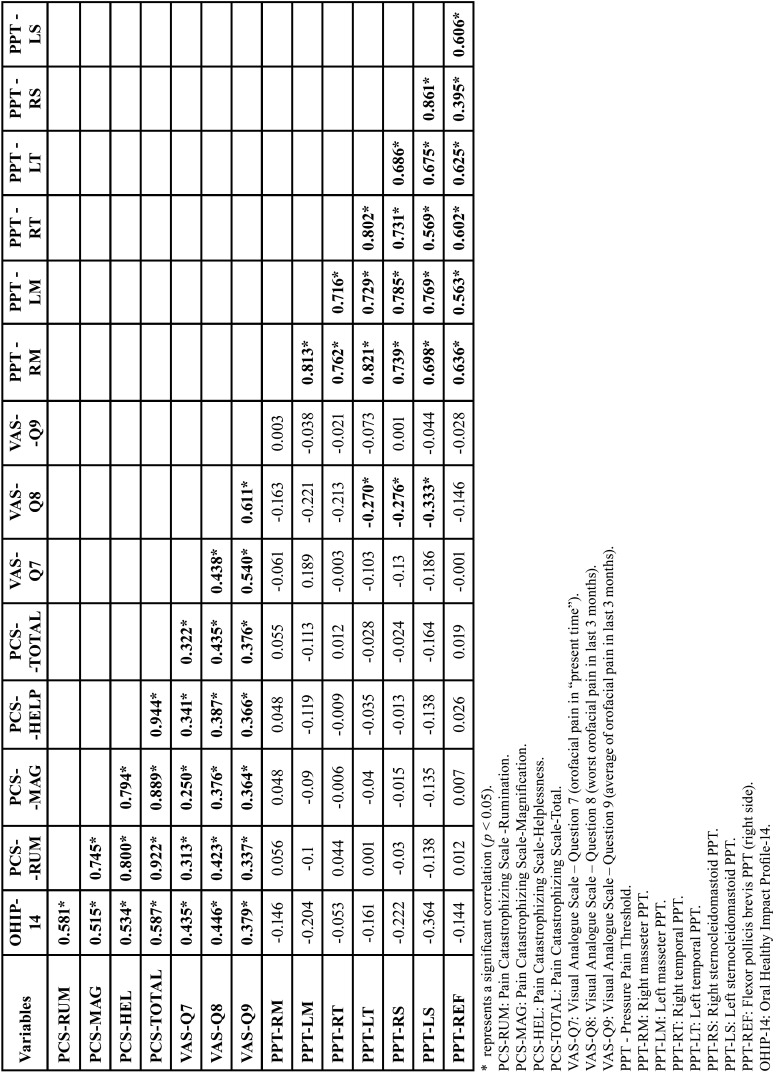
Spearman correlation between the variables studied in TMD group (n=97).

Table 3 presents the results of logistic regression to verify the influence of PCS domains on self-reported pain in TMD group. The rumination and magnification domains increased the chance of high self-reported pain in all situations (VAS-Q7, VAS-Q8 and VAS-Q9) (*p*<0.05). The helplessness domain only increased the chance of high self-reported pain for VAS-Q8 (*p*<0.05) (Table 3).

**Table 3:**
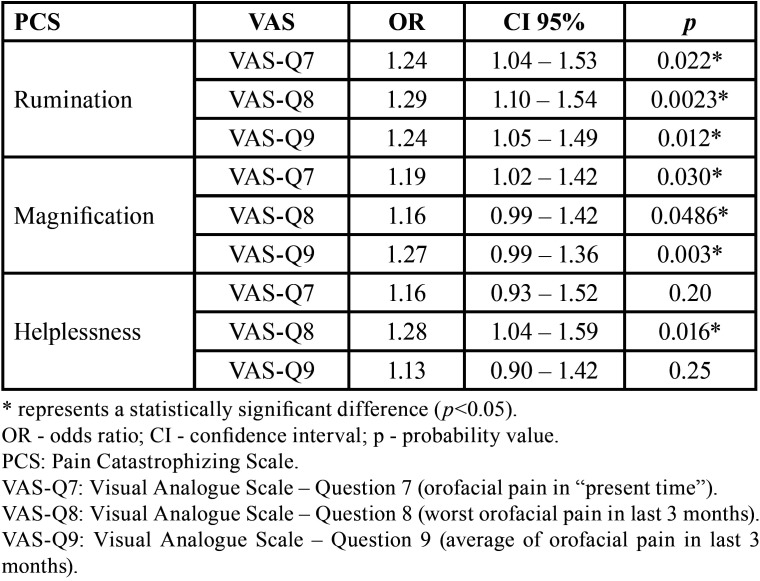
Logistic regression to verify the influence of PCS domains on self-reported pain in TMD group (n=97).

Table 4 shows only the statistically significant results of the logistic regression test (stepwise interactive procedure). Thus, the presence of TMD of muscular origin, high self-reported pain (VAS-Q7) or pain catastrophizing increased the chance of a low quality of life in relation to the control group (*p*<0.05). In addition, the reduction in sternocleidomastoid PPT increased the chance of poor quality of life (*p*<0.05) (Table 4).

**Table 4:**
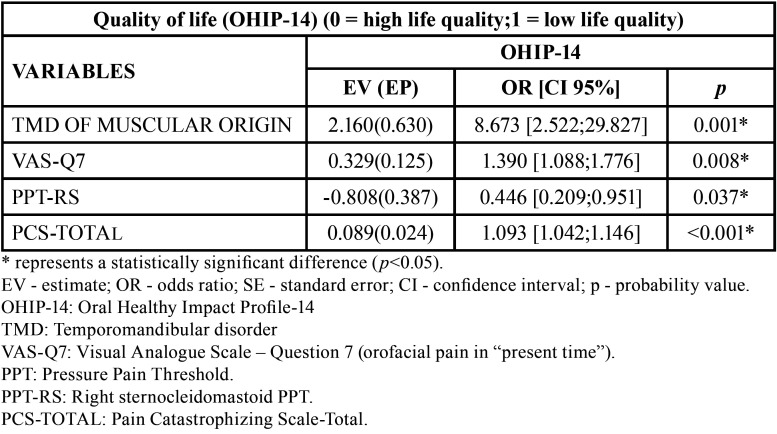
Logistic regression to verify the influence of studied variables on quality of life (OHIP-14) (n=194).

## Discussion

Most TMD subjects in this study were women (Table 1). This result corroborates other studies that also observed this situation (3,18). Hormonal (e.g., estrogen) and psychological factors (e.g., stress) may be causes of TMDs (19) and may explain the higher prevalence of women with TMD in TMD group, as women have higher estrogen levels than men and are more influenced by stressors than men (20).

TMD manifests most often in young and middle-aged individuals between 20 and 50 years of age (19). Table 1 shows that the mean age in TMD group was within this range of 20 to 50 years (43.3 years).

TMD group showed higher values of PCS (PCS-Helplessness, PCS-Magnification, PCS-Rumination and PCS-Total) and OHIP-14 than the control group (Table 1). This shows that TMD patients had higher levels of pain catastrophizing in all domains of the PCS scale, in addition to a lower quality of life compared with the control group (Table 1). Pain catastrophizing may have causal importance in the development and persistence of TMD-related pain (21). According to Turner *et al*., “catastrophizing is related to anxiety (i.e., anxiety is associated with the tendency to overemphasize the probability of a catastrophic outcome and the possible consequences of such an outcome)” (22). Thus, for patients with chronic pain (TMD group), the impact of anxiety on pain is related to central sensitization of nociceptive neurons, which contribute to the worsening of pain symptoms (23).

Table 2 shows that there was a significant positive correlation between self-reported pain (VAS-Q7, VAS-Q8 and VAS-Q9), pain catastrophizing (PCS-Helplessness, PCS-Magnification, PCS-Rumination and PCS-Total) and quality of life (OHIP-14). Therefore, the greater the self-reported pain, the greater the catastrophizing of pain and vice versa; the greater the self-reported pain, the greater the negative impact on quality of life (lower quality of life) and vice versa; and the greater the catastrophizing of pain, the greater the negative impact on quality of life (lower quality of life) and vice versa. It is important to note that the higher the OHIP-14 score, the lower the patient’s quality of life. In addition, there was a significant negative correlation between self-reported pain (VAS-Q8) and the PPT of the temporal (left) and sternocleidomastoid (both sides) muscles (Table 2). Thus, the higher the worst self-reported pain (in the last 3 months), the lower the muscle PPT and vice versa.

Although the correlations observed in the previous paragraph are significant, it is important to interpret the correlation coefficient (Table 2). Schober *et al*. reported the following interpretations for the correlation coefficients: 0.00–0.10 - Negligible correlation; 0.10–0.39 - Weak correlation; 0.40–0.69 - Moderate correlation; 0.70–0.89 - Strong correlation; and 0.90–1.00 - Very strong correlation (24). Thus, the correlations reported in the previous paragraph were, in most cases, considered negligible correlations and, in the minority of times, considered weak correlations (Table 2).

The TMD group showed significantly lower PPT values for all muscles studied (both sides) compared with the control group (Fig. 2). In addition, for the flexor pollicis brevis, the TMD group showed a significantly lower PPT value than the control group. This possibly occurred because the patients in the TMD group had chronic pain. This type of patient has central sensitization and this factor is probably important for a reduction in the muscle pain threshold (25).

Patients in the TMD group had pain in the masseter and temporal muscles due to TMD, in addition to pain in the sternocleidomastoid (cervical muscle). The relationship between the mandibular system (trigeminal nerve) and the cervical spine (spinal nerves) can be explained by the neuroanatomical convergence of nociceptive neurons that receive sensory input from the trigeminal and neck (12,25,26). Thus, disease in one of these systems can induce pain and/or dysfunction in the other system (12,25,26).

Logistic regression (Table 3) showed that all PCS domains can increase (*p*<0.05) the chance of worse self-reported pain (except between Helplessness and VAS-Q7 or VAS-Q9). This is clinically important, as it demonstrates the need to associate TMD treatment with cognitive-behavioral treatment through the coping strategy. According to Turner *et al*., “cognitive-behavioral theory posits that, for people with chronic pain, their attitudes and beliefs about their condition, as well as their behaviors, can influence their physical and psychosocial adjustment” (27) Thus, the effect of treatment on pain can be positively influenced in part when there is a pain coping response by the patient (27).

A low oral health-related quality of life due to TMD can be represented, for example, by pain, difficulty and inability to perform daily activities, irritation, tension, unsatisfactory diet, and difficulty speaking, eating and relaxing (15). The presence of TMD of muscular origin, high self-reported pain (VAS-Q7) or pain catastrophizing increased the chance of poor quality of life (*p*<0.05) (Table 4). It is noteworthy that TMD of muscular origin increased the chance of poor quality of life by 8.673 times. Thus, these factors (TMD, pain and pain catastrophizing) can work together to reduce the quality of life of a patient.

Table 4 also showed that reducing the sternocleidomastoid PPT increased the chance of poor quality of life (*p*<0.05). This result is important so that the dentist does not focus only on the masticatory muscles during TMD treatment. Thus, it is also important to treat neck muscle pain to improve the patient’s quality of life. For this, it is possible to use manual therapy. Manual therapy in masticatory and cervical muscles can trigger neurophysiological mechanisms responsible for pain relief and reduction in muscle activity, and this can improve the function of the region of interest (25).
